# A Bill to Regulate the Practice of Medicine in Texas

**Published:** 1893-01

**Authors:** 


A BILL,

To be Entitled An Act to regulate the practice of medicine and to prescribe the qualifications of Physicians and Surgeons, and to repeal Title No. LXXIII of the Revised Statutes.

SECTION 1. *Be it enacted by the Legislature of the State of Texas*, That no person shall hereafter be entitled to pursue the occupation of physician or surgeon, or to practice medicine or surgery, in any of their branches, in this State, without having first complied with the requirements hereinafter prescribed.

SEC. 2. The Governor, Attorney General, State Health Officer, President of the Board of Regents of the State University, and Dean of the Faculty of the Medical School of the University, shall constitute a board to be known as "The Board of Medical Control." Said board shall be presided over by the Governor, or in his absence, by the President of the Board of Regents. The Secretary of State shall act as its Secretary; its official acts shall be attested by him under the seal of State, and a majority of members shall compose a quorum. They shall meet as soon as practicable after the passage of this act, and thereafter at least once in each year, or as often as they may be called together by the Governor for the performance of the duties herein prescribed; the meetings to be held at the capital of the State. The members of said Board shall receive as compensation for their services, while in attendance on and going to and from said meetings, the sum of $5 per day, and their actual traveling expenses.

SEC. 3. It shall be the duty of said Board, as soon as practicable thereafter, to prepare and have printed and published a complete list of the reputable schools of medicine in the United States and foreign States and countries, copies of which list, duly certified by the Secretary of State, shall be at once furnished the county clerks of the several counties in the State, to be by them preserved for reference as hereafter provided. Said lists shall be so prepared and furnished the county clerks not later than October first of each year and as often as may be necessary to accomplish the purpose herein contemplated.

SEC. 4. By "reputable school of medicine" as herein named is meant one whose regular course of instruction comprises at least three full terms or sessions of at least six months each, and which is otherwise recognized and accredited as reputable by the school of medicine to which it belongs.

SEC. 5. Every person who desires to pursue the occupation of physician or surgeon, or to pracice medicine or surgery in any of its branches, shall, before entering upon the same, first have received a full diploma of graduation in due course from some reputable school of medicine, as the same is herein defined, and as listed by said Board of Medical Control, after having pursued the full three years course of instruction, exclusive of special or summer courses, or such proportion thereof as is required by such school before granting its diploma of graduation.

SEC. 6. Before entering upon the practice of medicine or surgery in any of its branches, the person desiring so to do shall exhibit to the clerk of the county in which he intends to pursue said practice, or of any county in which he may for the time reside, his diploma, as required in Section 5 hereof, and the clerk, upon inspection thereof and comparison of the same with the list of reputable medical colleges furnished him by the said board, shall, if satisfied of the genuineness and regularity of said diploma, and that the same was duly and legally issued to the holder of the same, issue to him a certificate reciting said facts, duly certified by the seal of his office, retaining in a well-bound book, kept in his office for that purpose, an entry of said certificate, showing the date of its issuance, the name of the person to whom it was issued, the name and location of the medical college from which the diploma was granted, and the date of its issuance. Certificates issued in accordance with this section shall entitle the holder thereof to practice medicine or surgery in any county in the State, and the county clerk shall receive a fee of two dollars and a half for each certificate so issued, to be paid by the person receiving the same. In case the person holding such certificate shall remove into any county other than the one in which the same was originally obtained, he shall, before entering upon the practice of his profession, have his certificate duly recorded in the clerk's office of the county into which he may have so removed, and in which he resides or sojourns; and the several county clerks shall keep in their offices well-bound books for that purpose, returning the original certificates to the holders thereof, with their certificates, under seal of recording, and for this service the clerks shall receive a fee of one dollar for each certificate recorded by them, to be paid by the person holding the same.

SEC. 7. All temporary certificates that may have been granted to persons by examining boards under the existing laws, shall be void upon the expiration of the period limited thereon, and in order to continue the practice of medicine or surgery in any of its branches, the holders of such temporary certificates are required to comply with the provisions of this act.

SEC. 8. The provisions of this act shall not be deemed to apply to the following persons:

1st. To those who may have already qualified for the practice of medicine under the provisions of an act entitled "An Act to regulate the practice of medicine," passed August 21, 1876, and amendments thereto.

2d. To those who have been regularly engaged in the general practice of medicine in the State, in any of its branches or departments, for a period of five consecutive years prior to January 1, 1875.

3d. Any female who practices midwifery strictly as such.

SEC. 9. No person except those named in the preceding sections of this act shall be permitted to practice medicine or surgery in any of their branches or departments in this State without having first obtained a certificate from a county clerk of some organized county of the State in accordance with the requirements provided in the foregoing portion of this act, and any person so attempting or professing to practice said occupation, shall be punished as provided in the penal code.

SEC. 10. Title LXXIII, of the Revised Statutes of the State of Texas, and all laws and parts of laws in conflict herewith, are hereby repealed.

Amend articles Nos. 396 to 399, of the penal code, so as to conform to above.

Also add article 3981, punishing any person who shall pass a bogus diploma, or one not issued to him, in order to obtain a certificate.

